# Supplementary material for: The causal relationship between the human gut microbiota and pyogenic arthritis: a Mendelian randomization study
Source: Front Cell Infect Microbiol. 2024 Nov 26;14:1452480. doi: 10.3389/fcimb.2024.1452480 (PMC11629706; doi:10.3389/fcimb.2024.1452480)
Supplement: Supplementary file 1 [file DataSheet1.zip › mendelian test/ebi-a-GCST90027552.csv_forest.pdf]

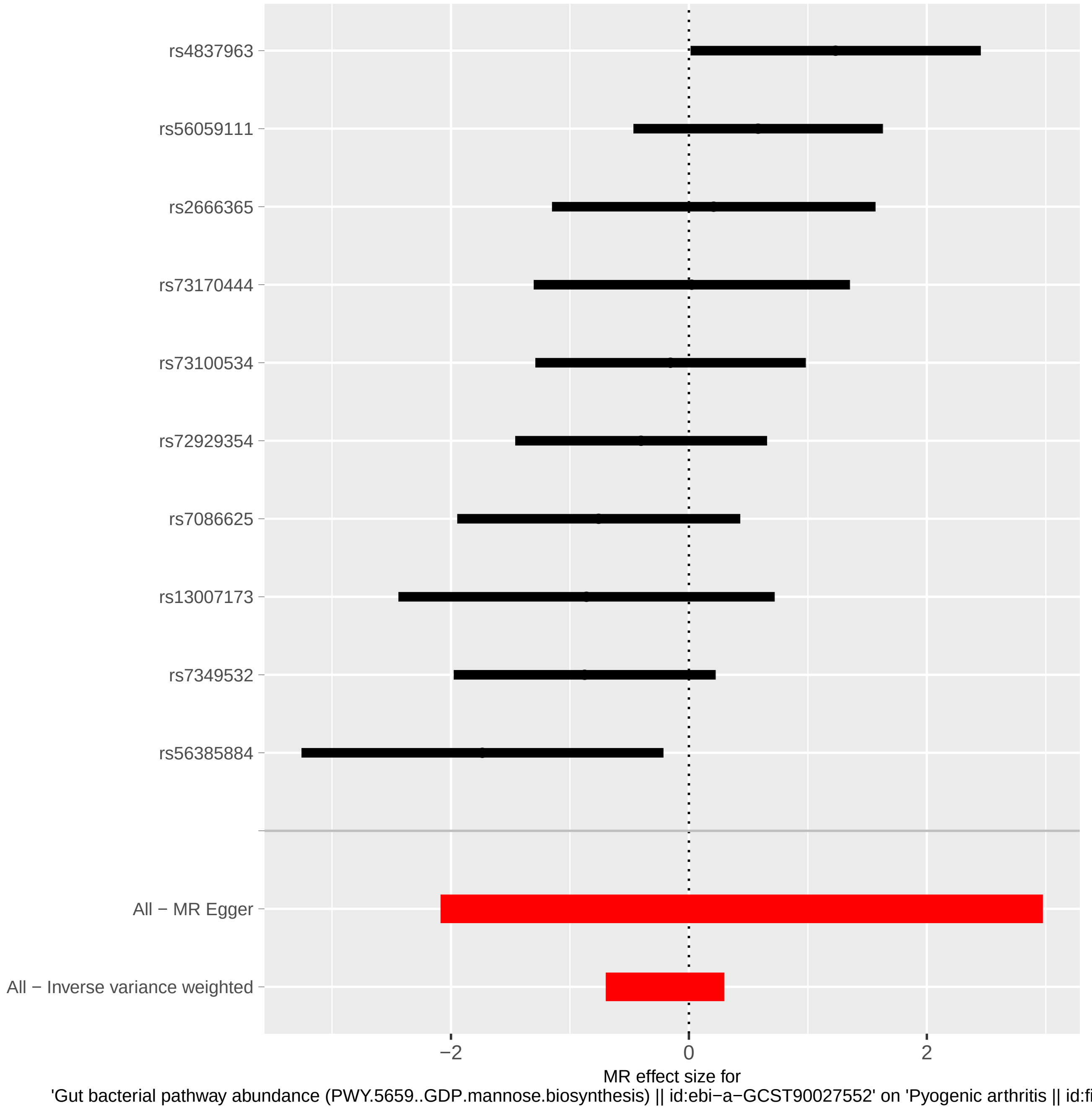
rs4837963
rs56059111
rs2666365
rs73170444
rs73100534
rs72929354
rs7086625
rs13007173
rs7349532
rs56385884
All − MR Egger
All − Inverse variance weighted
−2
0
2
MR effect size for
'Gut bacterial pathway abundance (PWY.5659..GDP.mannose.biosynthesis) || id:ebi−a−GCST90027552' on 'Pyogenic arthritis || id:fi
